# Autoimmune processes in neurological patients are much more common than presently suspected

**DOI:** 10.1007/s00415-023-11901-0

**Published:** 2023-08-21

**Authors:** Angelika Goertzen, Abdul Kareem Altawashi, Julian Rieck, Rüdiger W. Veh

**Affiliations:** 1 AMEOS Klinikum St. Clemens Oberhausen, Wilhelmstrasse 34, D-46145 Oberhausen, Germany; 2 Institut für Zell- und Neurobiologie, Centrum 2, Charité - Universitätsmedizin Berlin, Charitéplatz 1, D-10117 Berlin, Germany

**Keywords:** Autoimmune encephalitis, Cerebrospinal fluid (CSF), Autoantibodies, Immunocytochemistry, Hippocampus, CA2 area

## Abstract

Autoimmune encephalitides are seldom diseases. How rare they actually are, however, is not known. The low incidence combined with the problematic identification may dampen efforts of neurologists, to identify patients with unclear symptoms as suffering from autoimmune encephalitis. Here, we aim to obtain a better estimate, how many patients with autoimmune disorders should be expected among 100 inpatients in a conventional neurological department. From a total number of 2603 non-stroke patients attended in a 2-year period (2018–2019) 460 CSFs were obtained. From this collection 187 samples (40.7%, > 500 sections) could be analyzed with our immunocytochemical technique. Autoreactive antibodies were detected in 102 (55%) of these 187 CSF samples. Certainly, the presence of autoreactive antibodies does not necessarily indicate that the patient suffers from an autoimmune disease. Our data indicate that from roughly 2000 patients during 1 year about 125 patients with autoreactive CSF antibodies should be expected in a conventional neurological department. This represents the about 35-fold value of what is generally expected at present. Being aware of this high incidence may intensify the efforts of neurologist to identify patients with any type of autoimmune encephalitis. This will be beneficial for patients, because they often profit from immunomodulatory therapy. Interestingly, some CFSs from our patients react with the CA2 subdivision of the hippocampus. While long neglected, recent research places this area into an important position to influence hippocampal network physiology. Autoreactive antibodies in the CSF may disturb the function of CA2 neurons, thereby explaining some neuropsychiatric symptoms in patients with autoimmune encephalitides.

**Supplementary Information:**

The online version contains supplementary material available at 10.1007/s00415-023-11901-0.

## Introduction

During recent years, autoimmune encephalitides have been increasingly appreciated as nosologic entities in clinical neurology. The first subtype, anti-NMDA receptor encephalitis was described about 15 years ago [1] and has become one of the most important differential diagnoses for new-onset psychosis. Since then, quite a number of additional subtypes have been added including those based on autoantibodies against (1) AMPA receptors, (2) the voltage gated potassium channel complex (VGKC-complex; actually most antibodies are directed against LGI1 or CASPR2 proteins), (3) GABA-A and GABA-B receptors, (4) glycine receptors, and (5) the IGLON5 protein [2]. The incidence of autoimmune encephalitides has shown a threefold increase during the last 10 years [3], most likely attributable to increased identification of autoantibody-positive cases.

As might have been expected when a large variety of different antibodies potentially are involved, autoimmune encephalitides may clinically present as bouquet of symptoms. Many of them are also common in other neurological diseases, rendering direct diagnosis difficult. Consequently, such patients often are misdiagnosed [4] as psychosis or dementia, as movement disorder, as psychogenic behavior, or as conventional epilepsia, when suffering from intractable seizures [2]. Rapid progress of symptoms or anomalies in MRT images could be taken as indication for an autoimmune process. Final diagnoses, however, relies on the unequivocal demonstration of autoreactive antibodies [5].

This is achieved easily, when the corresponding antigen is present on the commercially available biochip (Euroimmun, Groß Grönau, Germany). It contains human embryonic kidney cells transfected with plasmids, which encode a restricted number of antigens commonly involved in autoimmune encephalitides. Quite a number of patients, however, present with signs, which strongly indicate an ongoing autoimmune process, but the biochip remains negative. This fact is not surprising, as there are only about 40 different antigens on the chip, while the mammalian brain comprises about 13,000 proteins. A certain amount (estimated 10%) of these will lose antigenicity subsequent to fixation. Approximately 11,500 proteins, however, will remain available, when fixed mammalian brain sections and immunocytochemistry are used for the detection of autoreactive antibodies in the CSFs of patients.

Autoimmune encephalitides might be considered as seldom diseases, but it is not completely known, how rare these disorders actually are. In Germany the incidence is thought to be 8 to 15 patients among 1 million inhabitants per year [6] and no other data are available. Actual figures, however, may be much higher, because the given incidence is based on fully diagnosed patients only, leaving the others with suspected and very likely autoimmune disorders aside.

In the clinical situation, the low incidence combined with the problematic identification may potentially dampen efforts, to identify patients with unclear symptoms as suffering from autoimmune encephalitis. A better estimate, how many patients with autoimmune disorders per year should be expected among 100 in-patients in a conventional neurological department, will be helpful for the neurologist to intensify efforts in identifying such individuals. This will be beneficial for patients, because they often profit from immunomodulatory therapy [7, 8].

Consequently, the present investigation aimed to obtain such an improved estimate. For this purpose, about 40% CSFs of all lumbar punctures in a conventional neurological department were analysed for the presence of autoreactive antibodies. As rat brain proteins show about 89–99% sequence identity to human ones, fixed rat brain sections were used as target for immunocytochemistry. As may have been expected, quite a number of immunoreactive CSFs were obtained from so far unsuspicious patients. One has to bear in mind, however, that the detection of autoreactive antibodies in any CSF does not necessarily mean that they interfere with the function of the target protein and that the corresponding patient suffers from an autoimmune disorder.

## Methods

### Chemicals

Chemicals were obtained from Sigma-Aldrich GmbH, Taufkirchen, Germany, if not indicated otherwise. Biotin labeled secondary antibodies and the Elite ABC complex were from Vector (Vector Laboratories, Burlingame, CA, USA).

### Brain tissue blocks

Rats were deeply anaesthetized and fixed via transcardial perfusion with a solution consisting of 4% paraformaldehyde, 0.05% glutaraldehyde, and 0.2% picric acid in 0.1 M phosphate buffer, pH 7.4 [9]. Brains were removed, cryoprotected in 0.4 M sucrose for about 4 h and in 0.8 M sucrose overnight, cut into blocks at preselected rostrocaudal levels, shock-frozen in hexane at − 70 °C, and stored at − 80 °C until use as described previously [10].

### Cerebrospinal fluids

Cerebrospinal fluids were obtained by lumbar puncture for diagnostic reason. Samples were tenfold diluted down to about 3 µg/ml IgG to avoid the detection of natural autoantibodies.

### Immunocytochemistry

For immunoperoxidase immunocytochemistry, freely floating coronal brain cryostat sections (25 mm) were subjected to immunocytochemistry as described earlier [10]. In short, sections were rinsed in phosphate buffered saline (PBS) and treated for 15 min with 1% sodium borohydride in PBS to remove residual aldehyde groups from the fixative. Sections were pretreated for 30 min in a blocking and permeabilizing solution (10% normal goat serum in 0.3% Triton X-100 and 0.05% phenylhydrazine in PBS at room temperature (RT). CSFs were applied for 36 h at appropriate dilutions in PBS containing 10% NGS, 0.3% Triton X-100, 0.1% sodium azide, and 0.01% thimerosal at 4 °C. Sections were thoroughly rinsed in PBS, pretreated for 1 h with PBS-A, and exposed for another 24 h at RT to the secondary antibody, diluted 1:5000 in PBS-A containing 0.1% Triton X-100. After repeated washings in PBS and preincubation for 1 h in PBS-A, the Elite avidin–biotin–peroxidase-complex (1:200 dilution in PBS-A) was attached to biotinylated secondary antibodies for another 12 h at RT. After additional rinses in PBS, preincubation for 15 min in a solution of 0.5 mg/ml diaminobenzidine, 3 mg/ml ammonium nickel sulfate, and 10 mM imidazole in 50 mM Tris buffer, pH 7.6, the visualization of the antigen–antibody complexes was started by the addition of 0.0015% hydrogen peroxide and stopped after 15 min at RT by repeated washings with PBS. Sections were mounted onto gelatine-coated slides, air-dried not longer than 30 min, dehydrated through a graded series of ethanol, transferred into xylene, and coverslipped with entellan.

### Nissl stain

Sections are mounted from PBS on gelatin-coated glass slides and dried for 30 min at RT. Subsequently they were left in 70% ethanol overnight, rinsed in bidistilled water, and stained with cresyl violet (0.2% cresyl violet acetate in 20 mM acetate buffer, pH 4.0) for 30 min at RT [11]. After rinsing in bidistilled water, sections were dehydrated fast through a graded series of ethanol, transferred into xylene, and coverslipped with Entellan.

### Study approval

All animal experiments were approved by the Regional Berlin Animals Ethics Committee and conducted in strict accordance with the European Communities Council directive regarding care and use of animals for experimental procedures. Adult male Wistar rats, weighing 250–300 g were obtained from our institutional breeder (Department for Experimental Medicine (FEM), Charité University Medicine Berlin). Animals were housed in group-cages under controlled temperature (22 °C) and illumination (12-h cycle) with water and food ad libitum. All clinical investigations were conducted according to Declaration of Helsinki principles. Written informed consent was received from participants at the Charité or the university Jena, Departments of Neurology, or their representatives prior to inclusion in the study and analyses were approved by the Charité University Hospital Institutional Review Board.

## Results

### CSFs from patients with suspected disorders, which had been excluded by laboratory findings, may serve as negative controls for immunocytochemistry

To obtain material for negative controls, CSFs from patients, who had undergone lumbar puncture to exclude disorders, such as borreliosis, meningitis, multiple sclerosis, or organopathological reasons for dysbehavior, were subjected to immunocytochemistry. These criteria were met by 26 patients, but 8 of them unexpectedly displayed positive immunoreactivity. Consequently, 18 CSFs could serve as negative controls for immunocytochemistry (Supplemental Fig. 1). None of these 18 CSFs displayed any immunoreactivity.

### CSFs from patients with a recognized autoimmune disorder, anti IgLON5 encephalitis, serve as positive controls for immunocytochemistry

**Fig. 1:**
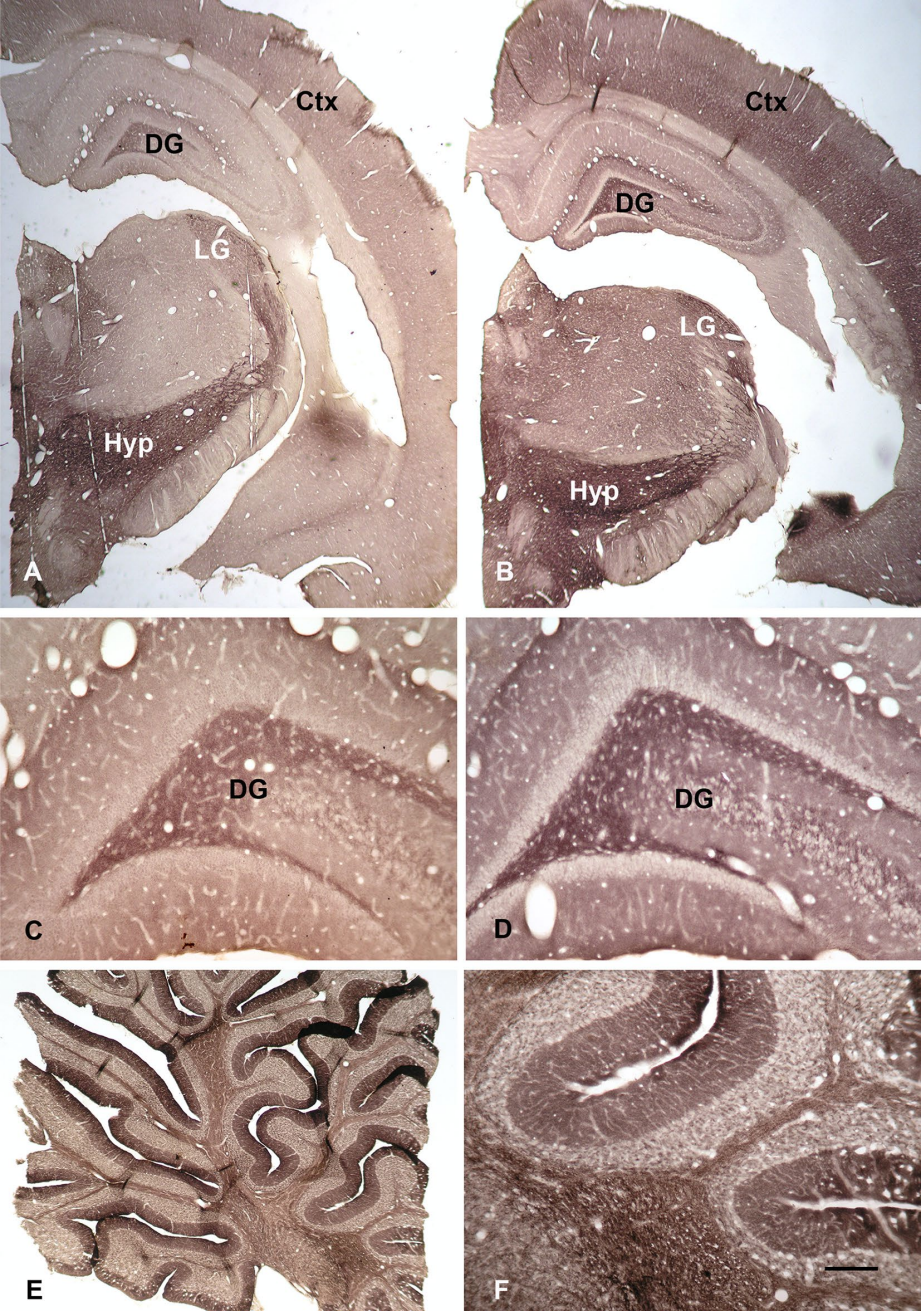
CSFs from patients with recognized anti IgLON5 encephalitis serve as positive controls in immunocytochemistry. Coronal sections through rat brains at the level of the hippocampus were treated with the CSFs from two patients (J-17_01 and J-17_17) with documented anti-IgLON5 encephalitis. The survey images **A**, **B** already document the closely similar staining patterns obtained with the two different CSFs. Note positive staining in cerebral cortex (Ctx), hilus of the dentate gyrus (DG), lateral geniculate (LG), and most strongly hypothalamus (Hyp) with both CSFs (**A**, **B**). This similarity is even more obvious after analysis of the dentate gyrus hilus at higher magnification (**C**, **D**). In the cerebellum, the molecular layer of the cortex (**E**) and the glomerula in granule cell layer **F** display strong immunoreactivity, the typical staining pattern produced by anti IgLON5 immunoreactivity [14]. Bar in **F** indicates 700 µm in (**A**) and (**B**), 150 µm in (**C**), (**D**) and (**F**), and 500 µm in (**E**)

Two of our patients (J-17_01 and J-17_17) were diagnosed by laboratory findings (Eurimmun, Lübeck) as suffering from anti-IgLON5 autoimmune encephalitis. Expecting to obtain the known staining pattern of IgLON5 we used these CSFs for immunocytochemistry with sections of rat forebrain and cerebellum. Indeed, both CSFs produced a closely identical staining pattern with positive cerebral cortex, hilus of the dentate gyrus, lateral geniculate, and most strongly the hypothalamus (Fig. 1A–D). Images are very similar to those shown earlier [12, 13], confirming the diagnosis as anti-IgLON5 autoimmune disease. In addition, the staining of the cerebellum (Fig. 1E, F) with a strongly positive molecular layer and clearly labeled glomerula in the granule cell layer also presents the typical anti-IgLON5 pattern [14]. These results indicate that our immunocytochemical technique is not only specific and highly sensitive, but even results in staining patterns characteristic for distinct autoimmune diseases.

### The incidence of autoimmune encephalitides in neurological departments may be much higher than previously recognized

**Fig. 2:**
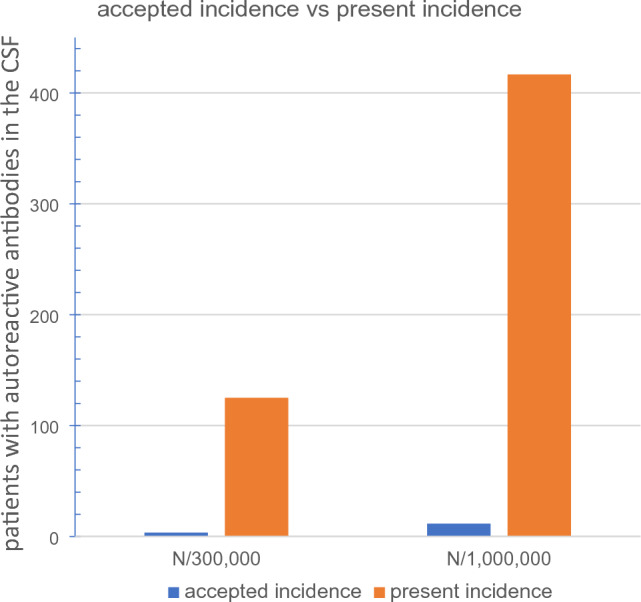
Incidence of patients with autoreactive antibodies is about 35-fold higher than accepted presently. From a total number of 2,603 non-stroke patients in a 2-year period 460 CSFs were obtained in our hospital. From this collection 187 samples (40.7%, > 500 sections) could be analyzed, and in 102 of these 187 CSF samples (55% of total CSFs analyzed) autoreactive antibodies were detected. These data indicate that about 125 instead of 3.5 patients, which are presently believed [6], with autoreactive antibodies in their CSFs must be expected during 1 year in a conventional neurological department

Aiming to obtain an unbiased estimate, how many patients with autoimmune disorders should be expected among 100 in-patients, we saved CSFs of most lumbar punctures performed during a 2-year period (January 2018 to December 2019) in our conventional neurological department (former St. Josef-Hospital, today AMEOS Klinikum St. Clemens, Oberhausen, Germany). We attended a total number of about 2000 patients per year (2127 persons in 2018, 1834 persons in 2019). Correcting this figure for people suffering from stroke or intracerebral hemorrhage (732 persons in 2018, 626 persons in 2019), who never receive lumbar puncture, from a total number of 2603 patients 460 CSFs were obtained in the 2-year period. From this collection 187 samples (40.7%, > 500 sections) could be analyzed with our immunocytochemical technique. Among these, immunoreactive antibodies could be detected in 102 of these 187 CSF samples (55% of total CSFs analyzed). Assuming that this percentage holds for all 230 lumbar punctures per year it indicates that about 125 instead of 3.5 patients, which are presently believed [6], with autoreactive antibodies in their CSFs must be expected during 1 year in a conventional neurological department (Fig. 2).

In the surrounding area, from where patients come to our hospital, live about 300,000 people. Based on the presently accepted incidence values (8 to 15, average 11.5, patients with autoimmune encephalitides per year per 1 million inhabitants [6]) one could expect 2.4 to 4.5 (average 3.5) such patients per year. In contrast, our data now suggest a total number of about 125 patients in our hospital with autoreactive antibodies in their CSFs. This represents 35 times the amount, which is expected by the presently accepted incidence value (Fig. 2). Consequently, autoimmune encephalitides are much more common than anticipated before.

One has to bear in mind, however, that the detection of autoreactive antibodies in any CSF does not necessarily mean that the corresponding patient in fact suffers from an autoimmune disorder.

In the surrounding area, from where patients come to our hospital, live about 300,000 people. The columns show the presently accepted incidence for our surrounding area as backcalculated from available data [6] as compared to our data, and the same comparison for 1 million inhabitants (as given by Wandinger et al. and backcalculated from our data). Consequently, autoimmune encephalitides are much more common than anticipated before. One has to bear in mind, however, that the detection of autoreactive antibodies in any CSF does not necessarily mean that the corresponding patient in fact suffers from an autoimmune disorder.

### Autoreactive CSFs are found in patients with quite distinct clinical diagnoses

Being aware of the importance of negative controls in immunocytochemstry, CSFs from patients with Alzheimer dementia, with degenerative cerebellar disorders, with excluded borreliosis or meningitis or with excluded organopathological reasons for dysbehavior were expected to devoid of autoreactive antibodies and were intended to be used as control samples. In fact, CSFs of none of the six patients, where organopathological reasons for dysbehavior had been excluded, showed any positive immunoreaction. Surprisingly, however, one of the four patients with Alzheimer dementia, eight of ten with degenerative cerebellar disorders, and six of twelve after exclusion of borreliosis or meningitis displayed immunoreactive CSFs (see Table 1).

**Table 1:** Autoreactive antibodies detected in patients with common neurological diseases

Prospective disorder	Expected control group	Analyzed	Positive
Alzheimer dementia	Yes	4	1
Amyotrophic lateral sclerosis	Yes	4	3
Autoimmune encephalitis	No	15	12
Benign intracranial hypertension	Yes	7	2
Creutzfeld–Jacob disease	No	1	1
Degenerative cerebellar disorders	Yes	10	8
Exclusion of borreliosis, meningitis, etc	Yes	12	6
Psychosomatic symptoms	Yes	6	0
Guillain–Barré syndrome	No	6	6
Multiple sclerosis	No	33	23
Neuralgic shoulder amyotrophy	No	6	4
Normal pressure hydrocephalus	Yes	3	0
Plexus paresis	?	2	2
Polyneuropathy	?	20	6
Slowly developing dementia	Yes	18	10
Unconventional headache	Yes	12	8
Others (cranial nerve palsy etc.)	Yes	32	10

CSFs from patients with Alzheimer dementia, with degenerative cerebellar disorders, or with excluded borreliosis or meningitis were not expected to contain autoreactive antibodies and intended to be used as controls. Surprisingly, quite a number of these expected controls turned out to be immunopositive

The presence of autoreactive antibodies in the CSFs of patients with autoimmune encephalitis, multiple sclerosis, Guillain–Barré syndrome, neuralgic shoulder amyotrophy, or paraneoplastic polyneuropathy could have been expected (Table 1). Unexpectedly, however, also three of four patients with amyotrophic lateral sclerosis, eight of ten patients with degenerative cerebellar disorders, or ten of eighteen slowly developing dementia displayed positive autoimmune reactivity (Table 1). Even more surprising was the group of patients, who were admitted because of strong headache. Usually, these patients are diagnosed, receive the corresponding therapy, and subsequently are dismissed comfortable. When lumbar puncture was performed to exclude subarachnoid hemorrhage or meningitis, it turned out that eight of twelve of this patient group displayed CNS autoreactivity. This fact should be kept in mind when dismissing such patients.

### Among patients, clinically diagnosed as suffering from autoimmune encephalitis, CSFs react with quite distinct structures

The present investigation comprised 15 patients (Table 2), who were tentatively diagnosed as suffering from autoimmune encephalitis. Diagnosis was based on short time developing and progressive psychiatric symptoms after exclusion of other organic reasons. One patient (J-19_025) showed anti-GFAP (glial fibrillary acidic protein) and another one (J-18_047) anti-CA2 (carbonic anhydrase II) and anti-GQ1b ganglioside reactivity, while all others were negative in the biochip assay.

**Table 2:** Some neurological patients with clinically suspected autoimmune encephalitis display autoreactive antibodies in their CSFs

Patient	M/F	Age	Preliminary diagnosis	Therapy	Final diagnosis	Dismission	Biochip	IC	Ctx	CA1	CA2	CA3	DG
J-18_043	F	81	AE	Cortison	AE	Healthy	0	+ +	+ +	+ +	+ +	+ +	+ +
J-18_047	M	73	AE	Cortison plasmaphor	Bickerstaff enceph	Unaltered	a-GQ1b	+ + +	+ +	+ +	+ + + +	+ +	+ +
J-18_051	M	55	AE	Cortison IgG	Startle disease	Healthy	a-GyR	+ + +	+ +	+ +	+ + +	+ + +	+ +
J-18_053	F	65	AE	Cortison	AE	Healthy	0	+ + +	+ +	+ +	+ +	+ +	+ +
J-18_080	F	83	AE	Acyclovir	Viral encephalitis	Healthy	0	+	+	+	+	+	+
J-18_122	F	80	AE	Cortison	AE	Unknown	0	+	+	+	+	+	+
J-18_128	M	71	AE	Cortison IgG	IgLON5?	Passed away	0	+ + +	+ +	+ +	+ +	+ +	+ +
J-18_166	M	62	AE	Psychiatric	Mania	Psychiatry	0	+ + +	+ +	+ +	+ +	+ +	+ +
J-19_003	F	66	AE	Cortison	M. Alzheimer	Demented	0	+	+	+	+	+	+
J-19_025	F	65	AE	Acyclovir antibiosis	Encephalitis	Healthy	a-GFAP	+ ?	+	0	0	0	0
J-19_057	F	59	AE	Cortison plasmaphoresis	AE	Mild depression	a-NR1	+ + +	+ +	+ +	+ +	+ +	+ +
J-19_091	F	64	AE	IgG	Unclear	Passed away	0	+ + +	+ +	+ +	+ + +	+ +	+ +
J-19_121	M	73	AE	Cortison	AE	Improved	0	–	0	0	0	0	0
J-19_133	F	78	AE	Lorazepam gabapentin	Unclear	Healthy	0	–	0	0	0	0	0
J-18_188	F	35	AE	Transferred	Dementia	Psychiatry	0	–	0	0	0	0	0

Quite a number but not all neurological patients with clinically suspected autoimmune encephalitis display autoreactive antibodies in their CSFs. Furthermore, some of them show an increased immunoreactivity in the CA2-region, which is increasingly recog-nized as a functionally most important area in the hippocampus

*AE* autoimmune encephalitis, *IC* immunocytochemistry, *Ctx* cerebral cortex, CA1, CA2, CA3, *DG* hippocampal areas

CSFs from eight of these patients contained strongly autoreactive antibodies (Table 2, supplemental table) and five of them could be dismissed in healthy (4) or strikingly improved (1) condition. In six patients the diagnosis autoimmune encephalitis was maintained until dismission (Table 2). One of these patients (J-18_043) experienced a miraculous improvement subsequent to cortison therapy and we already had confirmed the presence of autoreactive antibodies in the corresponding CSF [15].

**Fig. 3:**
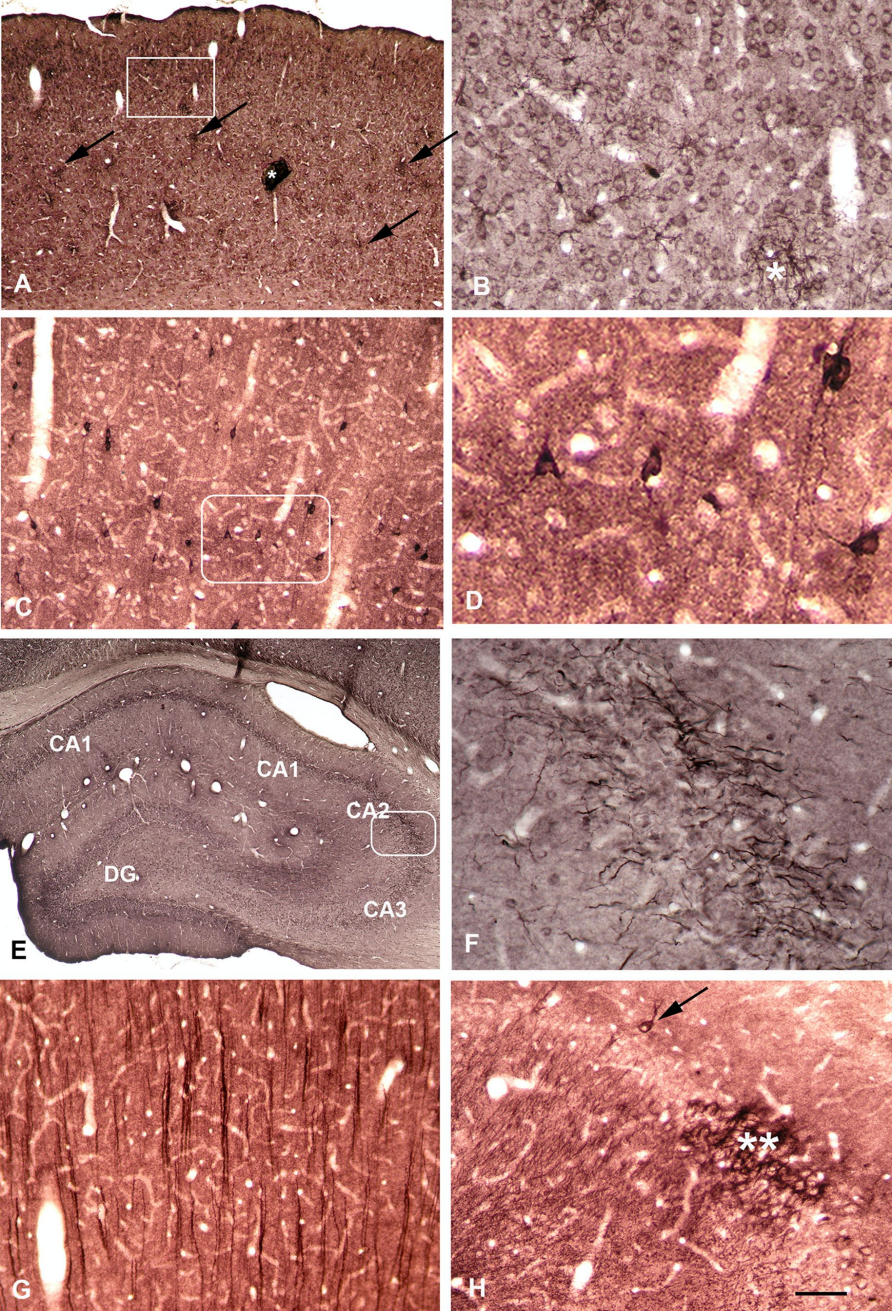
Autoreactive CSFs from patients perspectively diagnosed as suffering from autoimmune encephalitis display diverse structures in immunocytochemistry. Coronal sections through rat brains at the level of the hippocampus were subjected to immunocytochemical treatment with CSFs of four patients, which had been perspectively diagnosed as suffering from autoimmune encephalitis. **A** The CSF of patient J-18_043 reacts with neuronal cell bodies and astrocytes (boxed area at higher magnification in **B**). Note the patchy organization as visualized in the survey micrograph. Patches (exemplary arrows) represent either an increased density and staining intensity of neurons (**B**, compare upper right field to the lower left corner) or dense plexuses of neuronal processes (**B**, asterisks). **C** The CSF of patient J-19_091 predominantly interacts with interneurons and stains the neuropil (**C**, **D**). Boxed area in (**C**) is displayed at increased magnification in (**D**) and shows cell bodies and dendrites of heavily stained interneurons. **E** The CSF of another patient, J-18_051, in addition to CA1 neurons in the hippocampus **E** predominantly targets axons as already detectable in the survey micrograph (**E**, boxed area). Axon staining is most pronounced in the CA2 area (**F**). **G** In contrast, the CSF of patient J-18_047 yields intensely immunoreactive primary dendrites, mildly stained cell bodies of pyramidal neurons, and the neuropil in the cortex (**G**). In the hippocampus, however, the same CSF also reacts with dendrites, cell bodies, and the neuropil (**H**). Note the strongly and selectively increased immunoreactivity selectively in the CA2 (double asterisks) area of the hippocampus (**H**). Some interneurons also are stained strongly (H, arrow). Bar in **H** indicates 625 µm in (**A**), 125 µm in (**B**), 550 µm in(**C**), 150 µm in (**D**), 400 µm in (**E**), 60 µm in (**F**), and 300 µm in (**G**) and (**H**)

Not unexpectedly, immunocytochemical staining patterns of the positive CSFs were quite different. The CSF of patient J-18_043 reacts with neuronal cell bodies and astrocytes (Fig. 3). Distribution of cell bodies is not homogenous, resulting in striking dark patches already detectable in the survey micrograph (Fig. 3A). However, patches may also represent dense plexuses of neuronal processes (Fig. 3B, asterisks).

The CSF of patient J-19_091 shows interneurons with strongly immunoreactive cell bodies sticking out from the surrounding less positive neuropil (Fig. 3C, D). Staining comprises cell bodies and dendrites (Fig. 3D). Selectivity for interneurons is impressive, as other cortical neurons display weak if any immunoreactivity (Compare Fig. 2A–D).

The CSF of patient J-19_091 shows interneurons with strongly immunoreactive cell bodies sticking out from the surrounding less positive neuropil (Fig. 3C, D). Staining comprises cell bodies and dendrites (Fig. 3D). Selectivity for interneurons is impressive, as other cortical neurons display weak if any immunoreactivity (Compare Fig. 2A–D).

The CSF of another patient, J-18_051, targets pyramidal cell bodies in the hippocampus (Fig. 3E). In addition, it strongly displays terminal axons, especially in the hippocampal CA2 area with astonishing specificity (Fig. 3F).

In contrast, the CSF of patient J-18_047 yields intensely immunoreactive main dendrites and mildly stained cell bodies of pyramidal neurons in the cortex (Fig. 3G). In the hippocampus proper the same CSF also reacts with dendrites and cell bodies and a few interneurons (Fig. 3H, arrow). The neuronal staining pattern is strikingly pronounced selectively in the CA2 area of the hippocampus again (Fig. 3H). This area, which had been largely neglected until recently, is now known to be involved in social memory and neuropsychiatric diseases [16–19].

## Discussion

Diagnosing a patient as suffering from autoimmune encephalitis is a rare event, but it is not known, how seldom it actually is. Consequently, this investigation aimed to obtain an estimate about the percentage of patients with ongoing autoimmune processes in a conventional neurological department at present. Certainly, it is difficult to learn, how many patients suffer from an autoimmune disorder, when the unequivocal demonstration of autoreactive antibodies is mandatory for reliable diagnosis. Unfortunately, laboratory findings at present mostly rely on the commercially available biochip (Euroimmun, Groß Grönau, Germany). Due to its inherent mechanism this chip can detect only a restricted number (about 40) of antigens. Thus, patients with unknown autoantigens, which, therefore, cannot be present on the chip, will not be recognized by the conventional procedure.

To obtain a largely unbiased estimate, how many in-patients in a neurological department may suffer from any autoimmune encephalitis, the vital prerequisite is an appropriate assay, which detects most autoantibodies in the CSFs of neurological patients. In the present investigation, therefore, immunocytochemistry with fixed rat brain sections was used. This procedure is expected to detect about 11,500 distinct antigens via positive staining results. Certainly, immunocytochemistry is a capricious technique, requiring valid positive and negative controls.

### Technical considerations

Interpretation of immunocytochemical experiments strongly depends on appropriate controls. Human body fluids often contain natural autoantibodies, which usually are present in low concentrations only [20]. In previous work we also used immunocytochemistry with fixed rat brain sections to detect autoantibodies in the sera of patients with Guillain–Barré syndrome [21–23]. Sera were highly diluted (1:5000; down to about 2 µg/ml IgG) to avoid the detection of natural autoantibodies. In the present report, therefore, CSFs also were diluted down to a similar IgG concentration (3 µg|ml). This technique had recently been used to differentiate between two types of anti-NMDA-R1 encephalitis [24].

Here, CSFs from patients with excluded borreliosis, meningitis, multiple sclerosis, or organopathological reasons for dysbehavior were analysed as prospective negative controls (Table 1). A minor proportion of them, however, produced positive staining results. In these cases the reasons for the presence of autoreactivity remain unresolved. The CSFs of most of the other patients showed no autoreactivity and produced blank sections (Supplemental Figure). Based on these results it is concluded that our technique does not detect natural autoantibodies, and subsequent results are valid.

Furthermore, we used the CSFs of two different patients suffering from IgLON5 encephalopathy as positive controls (Fig. 1). Both CSFs produced close to identical staining, yielding the typical pattern of anti-IgLON5 antibodies [14], thereby supporting the high specificity of our procedure.

### The incidence of patients with autoreactive antibodies in the CSF is much higher than anticipated

Certainly, the incidence of autoimmune encephalitides in Germany appears rather low. Presently a value of 8 to 15 patients among 1 million inhabitants per year is accepted [6]. These data are based on the registry of The German Network for Research on Autoimmune Encephalitis (GENERATE e. V.). It was created as a multicenter study, focusing on patients with a clinical syndrome of autoimmune encephalitis [25]. Data from other countries are not available. The apparently low incidence, suggested by these data, combined with the difficulties in the unequivocal identification may potentially dampen efforts, to identify patients with unclear symptoms as suffering from autoimmune encephalitis.

Our study, in contrast, is not focused on any clinical symptomatology. Instead, it aimed to include any patient in a conventional neurological department, who received lumbar puncture by any reason. Using this approach, we aimed to detect without preoccupation any patient, who bears an ongoing autoimmune process. That is why our inclusion criteria are completely different from those used in the German registry.

Our data now suggest an incidence of 200 to 300 patients among 1 million inhabitants with autoreactive antibodies in their CSFs (Fig. 2). Certainly, one has to bear in mind that the detection of autoreactive antibodies does not necessarily mean that the corresponding patient suffers from an autoimmune disorder. Nevertheless, being aware of the high incidence of patients with autoreactive antibodies in the CSF will intensify the efforts of the neurologist to detect patients with any type of autoimmune encephalitis. This will be beneficial for patients, because they often profit from immunomodulatory therapy [7, 8].

### Autoreactive CSFs are found in patients with quite distinct clinical diagnoses

In line with the increased incidence of autoreactive antibodies, ongoing immune processes were recognized in quite a number of patients, suffering from other common neurological diseases (Table 1).

In cases of Guillain–Barré syndrome, multiple sclerosis, neuralgic shoulder amyotrophy, and few with polyneuropathic (vasculitis linked to collagenosis, paraneoplastic event, monoclonal gammopathia) the presence of autoreactive antibodies in the CSFs comes with no surprise. In multiple sclerosis, however, it is unexpected at the first glance that only 23 of 33 patients yielded a positive immunoreaction (Table 1). This may be explained by one of two simple facts. First, lumbar puncture in patients with prospective multiple sclerosis mostly is performed to ascertain diagnosis. At this early stage antibody level can be low and thereby escape detection. Second, one has to bear in mind that we work with fixed brain sections. Consequently, there will be epitopes, which become destroyed during fixation, and, therefore, cannot be visualized by our procedure.

In contrast, the detection of autoreactive antibodies in cases of amyotrophic lateral sclerosis, benign intracranial hypertension, degenerative cerebellar disorders, exclusion of borreliosis and others, non-Alzheimer dementia, plexus paresis, or unconventional headache was astonishing (Table 1). Certainly, the low number of patients in these groups precludes firm statements. The simple fact, however, deserves further investigation.

Satisfactorily, not all groups neurological patients displayed immunopositive CSFs. Thus, no patient with psychosomatic symptoms or with normal pressure hydrocephalus presented with autoreactive antibodies in the CSF (Table 1).

### CSFs from patients with autoimmune encephalitis react with quite distinct structures

In patients suffering from autoimmune encephalitis the presence of autoreactive antibodies comes not as surprise. So far, the NR1 subunit of NMDA receptors apparently often is involved [2, 5, 24, 26, 27]. Since the first description [1] quite a number of additional autoimmune encephalitides have been recognized [2]. It is not surprising, therefore, that CFSs from our group of patients with this disease react with quite distinct cellular structures in a number of different areas (Table 2). Thus, the CSF from patient J-18_043 results in heterogeneously distributed positive neurons and also axons in a patchy distribution (Fig. 2A, B), while that from patient J-19_091 predominantly shows one beautifully visualized class of interneurons (Fig. 2C, D). Most interesting, however, is the prominent staining of the hippocampal CA2 region (Fig. 2E, F, H).

The hippocampus proper presents as four subregions, CA1 (Cornu ammonis 1), CA2, CA3, and dentate gyrus (DG). The CA2 subdivision (Fig. 2E, supplemental figure) is a small region between CA1 and CA3, which had largely been ignored for a long time. CA1 and CA3 subregions together with the DG had been considered as most important functional areas. However, recently, a biological function other than that of CA1 and CA3 could be suspected for CA2. Its projections to CA1 and CA3 place it into a good position to influence hippocampal network physiology and information processing in the hippocampus [28, 29]. Several genes are differentially expressed [30]. The vasopressin 1b receptor is enriched in the CA2 area [31] and the deletion of its gene disrupts social recognition memory [16]. Furthermore, CA2 is affected in a number of neuropsychiatric diseases, such as schizophrenia and bipolar disorder [17, 18] including animal models [19]. In our experiments several autoreactive antibodies prominently stain the CA2 region (Fig. 2E, F, H) and most others also are positive (Table 2). These antibodies may disturb the function of CA2 neurons, thereby explaining neuropsychiatric symptoms in patients with autoimmune encephalitides.

### Immunocytochemistry to detect autoreactive antibodies is not appropriate for clinical routine diagnosis

Certainly, immunocytochemistry is a powerful method to detect autoreactive antibodies in bodily fluids, such as CSFs. Not appropriate, however, is it for clinical routine diagnosis. Consequently, additional research presently ongoing in our laboratory aims to develop alternate techniques. We expect that the novel procedures will provide the same sensitivity and specificity, which we have now, combined with an easy handling for the everyday clinical application.

### Supplementary Information

Below is the link to the electronic supplementary material.

Supplementary file1 (DOCX 5898 kb)

## Data Availability

The data sets used and/or analysed during the current study are available from the corresponding author on reasonable request.

## Abbreviations

AMPA: Alpha-amino-3-hydroxy-5-methyl-4-isoxazolepropionic acid
CA: Hippocampal cornu ammonis
CASPR2: Contactin-associated protein 2
CA2: Carbonic anhydrase II
CSF: Cerebrospinal fluid
DG: Hippocampal dentate gyrus
GABA: Gamma-aminobutyric acid
GFAP: Glial fibrillary acidic protein
GQ1b: Ganglioside Q1b
IGLON5: Immunoglobulin-like cell adhesion molecule 5
LGI1: Leucine-rich glioma inactivated 1
NMDA: N-methyl-d-aspartate
NR1: NMDA-Receptor subunit 1
PBS: Phosphate buffered saline
RT: Room temperature
VGKC: Voltage gated potassium channel
