# Supplementary material for: Autoimmune processes in neurological patients are much more common than presently suspected
Source: J Neurol. 2023 Aug 21;270(12):5866–77. doi: 10.1007/s00415-023-11901-0 (PMC10632246; doi:10.1007/s00415-023-11901-0)

Supplemental Figure: Negative controls for immunocytochemistry using CSFs from patients with suspected disorders, which had been excluded by laboratory findings

Coronal sections through rat brains at the level of the hippocampus were subjected to staining with cresyl violet (A) or to immunocytochemical treatment with CSFs of patients, which had received lumbar puncture to exclude inflammatory polyneuropathy (B, patient: J-18_192), Alzheimers disease (C, patient: J-19_041) or Borreliosis (D, patient: J-181_78).

CA1: hippocampal cornu ammonis 1, DG: hippocampal dentate gyrus. Bar in (D) indicates 300 µm in (A), (B), (C), and (D).


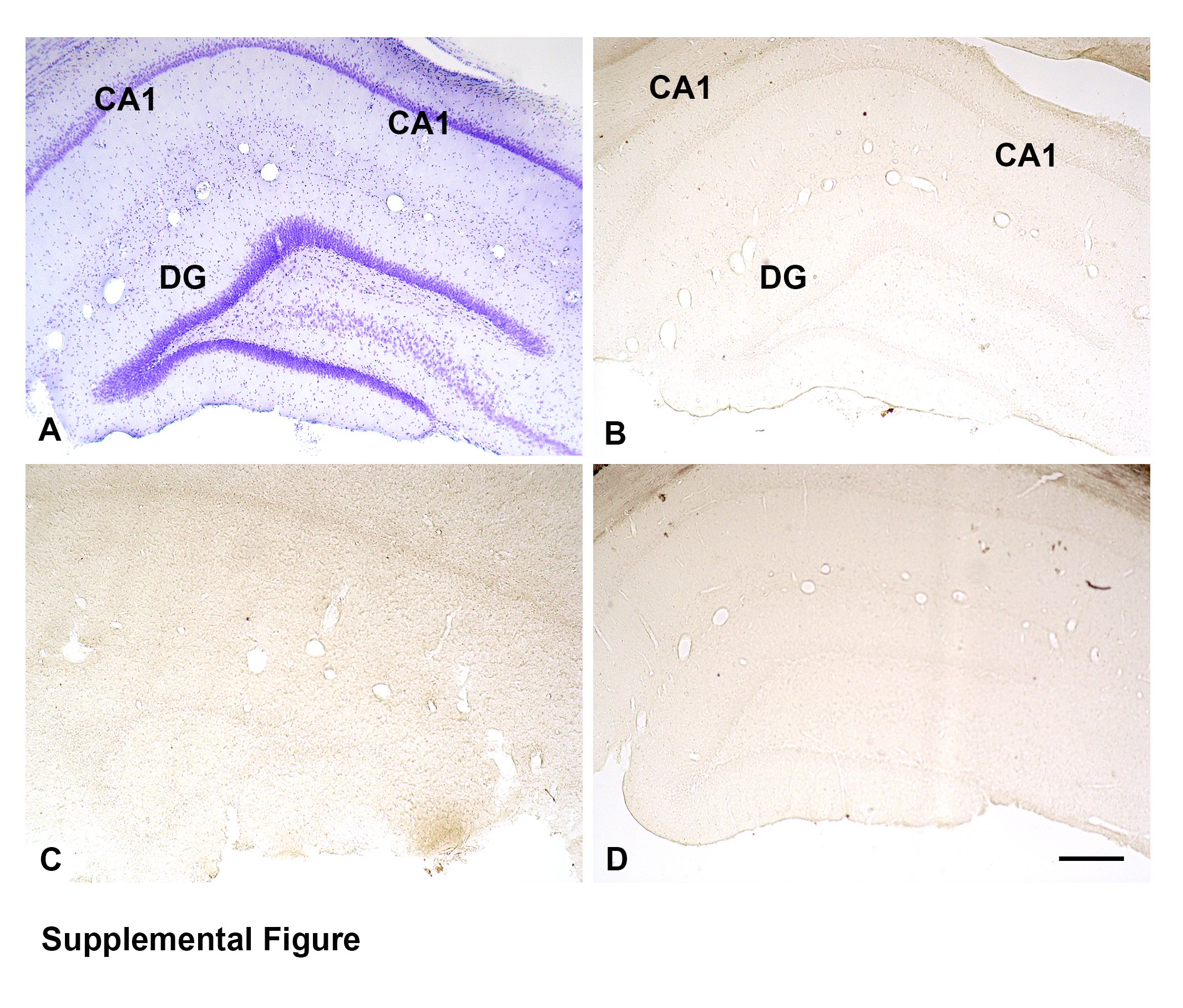

Supplement: Supplementary file 1 — Supplementary file1 (DOCX 5898 kb) [file 415_2023_11901_MOESM1_ESM.docx]
